# *TLR-8*, *TNF-α*, and *ESR-1α* Gene Polymorphism Susceptibility in Onset of Arthritis

**DOI:** 10.1155/2022/9208765

**Published:** 2022-09-20

**Authors:** Maryam Mukhtar, Nadeem Sheikh, Andleeb Batool, Tayyaba Saleem, Muhammad Babar Khawar, Mavra Irfan, Saira Kainat Suqaina

**Affiliations:** ^1^Cell and Molecular Biology Lab, Institute of Zoology, University of the Punjab, Lahore, Pakistan; ^2^Rhumatology Domain, Center of Molecular Medicine, Karolinska Institute, Department of Medicine, Stockholm, Sweden; ^3^Department of Zoology, Government College University, Lahore, Pakistan; ^4^Applied Molecular Biology and Biomedicine Lab, Department of Zoology, University of Narowal, Narowal, Pakistan

## Abstract

Arthritis is a genetic disorder characterized by bones and joint degradation assisted by severe pain and inflammation. It is evident by the studies that 0 candidate genes variations play vital role in its development and progression. Therefore, we investigated the genetic variation of *TLR-8*, *TNF*, and *ESR-1α* genes in the Pakistani population. A case-control study comprising 300 RA, 316 OA, and 412 control subjects was conducted. PCR-RFLP and direct sequencing methods were used for determining genetic variations. Analysis was performed by using PLINK and MEGA 6.0 software. Allelic and genetic frequencies of polymorphisms identified on rs3764879 (*TLR-8*), rs3764880 (*TLR-8*), rs5744080 (*TLR-8*), rs1800629 (*TNF*), rs2228480 (*ESR-1α*), and rs1451501590 (*ESR-1α*) were significantly varied among RA, OA, and controls. Novel functional mutations SCV000844945 and SCV000844946 on *TLR-8* as well as a non-functional SCV000804801 and functional variation SCV000804802 on *ESR-1α* were also identified and reported for the first time in the studied population. Multiple site analyses indicated that polymorphisms on *TLR-8* and *ESR-1α* genes were significant risk factors in disease onset to the next generation. In conclusion, *TLR-08* and *ESR-1α* were significant in the onset of arthritis whereas the *TNF* was not found as a significant risk factor in the onset of RA and OA.

## 1. Introduction

Rheumatoid arthritis (RA) and osteoarthritis (OA) both are bone disorders characterized by the mutual interactions of environment and genetics. However, RA is an autoimmune disorder whereas OA is non-autoimmune but in both diseases bones and joints get affected and degraded one way or another [1]. Innate and adaptive immunities enable the immune system to fight against multiple disorders. Innate immunity detects the presence of antigens through pattern recognition receptors (PRRs) recognized specific pathogenic molecules in microbes along with host endogenous ligands and leads to the pro-inflammatory cytokines, costimulatory molecules, and interferon (IFNs) expression [2,3]. The PRR was further classified into two major classes, i.e., cytosolic and transmembrane. Toll-like receptors (TLRs) are the first known transmembrane PRR. In humans, total of ten TLRs were classified on cell surfaces (*TLR-1, TLR-2, TLR-4, TLR-5, TLR-6,* and *TLR-10*) and in endosomes (*TLR-3, TLR-7, TLR-8*, and *TLR-9*) [4]*. TLR-8* functions like *TLR-7* as involved in the reorganization of self RNA, within snRNP autoantibodies complexes, viral RNA, and multiple small molecule agonists [5–10]. However, *TLR-8* is more likely activated by AU-rich ssRNA and senses it through its secondary structures [6, 11]. *TLR-8* expressed itself in myeloid DCs, monocytes, and neutrophils [6, 12, 13]. Human *TLR-8* got activated by the endogenous ligands might lead to the onset of many inflammatory diseases such as rheumatoid arthritis RA as well as OA.

Tumor necrosis factor-alpha (*TNF-α*), a pleiotropic and pro-inflammatory cytokine, is encoded by the *TNF-α* gene which is allocated on chromosome 6 : 31 [14]. In RA patients, its overproduction primarily by macrophages in joints causes the establishment of rheumatoid synovitis, formation of pannus tissue as well as the destruction of the joint. Its elevated level results in the increase of synoviocyte proliferation and leads to the activation of a secondary mediators' cascade, playing a role in inflammatory cell recruitment, joint destruction, and neo-angiogenesis [15]. It along with interleukin-1*β* (*IL-1β*) increased impaired cartilage function and stiffness of articular chondrocytes [16, 17]. In vitro studies indicated that pro-inflammatory *TNF-α* upregulates mRNA matrix metalloproteases-1 and -3 chondrocyte which leads to the pathogenic of OA as a potential risk factor [16–18]. *TNF-α* mRNA expression in knee OA patients was found to be 1.56 times greater as compared to controls [14].

Estrogen Receptor 1 (*ESR-1*) gene encodes for estrogen receptors (ERs), located on chromosome located on 6q25 [19]. The main determinant of bone strength is the cortical bone dimensions, and it is regulated by the estrogen receptor (ER) mediated and mechanical loading (see [20, 21]). It has been evident that estrogens function as a protector against bone matrix loss and are mediated by estrogen receptor-alpha (ER-*α*) [22], [23, 24]. It stimulates transcription through activation functions (AFs), i.e., AF-1 located on N-terminal and AF-2 located in the ligands binding domain [25]. ER-*α* activates the genes by the hormone-receptor binding which is then followed by gene activation with estrogen response element (ERE) having promoters. It increased the transcriptional activity from ERE-reporter transfected into osteoblast cell-line [26]. We have recently found that *PADI-4* gene polymorphism was not only involved in the onset of RA but was also found to be a significant risk factor in OA onset [1]. Both RA and OA are genetic disorders attributed to the environment and daily lifestyle, but the etiology of both diseases is still a mystery concerning the Pakistani population; therefore, the current study was designed to determine the polymorphism of *TLR-8*, *TNF*, and *ESR-1α* gene and its association with the onset of both RA and OA.

## 2. Materials and Methods

### 2.1. Ethical Approval and Sampling

The current study was approved by the ethical committee of the Department of Zoology, University of the Punjab, Lahore, Pakistan, and ethical committees of the government and semi-government hospitals of Punjab province of Pakistan from where samples were collected. All participants were already diagnosed with RA and OA by the physician. Blood samples (3 cc) were collected from each subject in EDTA coated tubes with BD syringes after taking informed written consent from the patient/guardian along with their clinical data. Blood samples were also collected from age and sex-matched healthy control subjects with a negative family history of arthritis. Samples were stored at 4^o^C before being further processed.

### 2.2. Genotyping

DNA was extracted from each blood sample of controls as well as patients manually by using a modified organic DNA extraction method [27] and was quantified (conc. ng/*μ*l) and qualified (260/280) by using Nanodrop. Three SNP's rs3764879, rs3764880, and rs5744080 on *TLR-8*; two SNP's rs1800629 and rs361525 on TNF, and four SNP's rs2234693, rs9340779, rs2228480, and rs1451501590 on *ESR-1α* were selected on basis of their either direct or indirect involvement in either the pathogenesis or progression of disease. For the amplification of targeted polymorphic sites, specific primers used are presented in Table 1. The polymerase chain reaction (PCR) was conducted in a 25 *μ*l reaction mixture consisting of 10 *μ*l of master mix (Thermo Scientific), 3 *μ*l of forward and reverse primer each, and 12 *μ*l of DNase, RNase-free water. The PCR conditions include initial denaturation at 94^o^C for 5 mins followed by 35 repeated cycles of denaturation at 94^o^C for 45 secs, annealing temperature presented in Table 1 for 45 secs, extension at 72^o^C for 30 secs, and then final extension at 72^o^C for 10 mins. The products were run on 2% agarose for confirming their product sizes. Genotyping was performed by restriction fragment length polymorphism (RFLP) and direct sequencing. For RFLP, PCR products were digested with their respective enzymes (Table 1) and were run on 2% agarose gel. For the direct sequencing method, PCR products were sequenced by using a sequencer, and sequences were visualized on BioEdit software.

### 2.3. Statistical Analysis

The genetic data for each subject was tabulated and passed through Hardy Weinberg Equilibrium (HWE) Test. Allelic and Genotypic Test was performed. The significance level was determined by the chi-square test and Fisher exact test. Linkage Disequilibrium and Haplotype analysis were performed fanalysesple site analysis. The change in the amino acid sequence was determined by MEGA 6.0 software.

## 3. Results

The mean age of RA males was 38.62 years and that of RA females was 38.98 years with a mean age of diagnosis in years was 33.06 and 31.56, respectively. Both RA males and females were in the normal BMI range, i.e., 18.75–25.5 kg/m^2^. The mean age of OA males was 55.27 years and for females was 49.19 years with a mean age of diagnosis in years was 46.97 and 42.73, respectively. In the OA group, obesity, i.e., BMI range of 25.5–30.5 kg/m^2^ was predominating.

It was observed that allele G on rs3764879 (*TLR-8*) was prevalent in patients whereas on rs3764880 (*TLR-8*) allele G got replaced by allele A in patients. On the rs5744080 (*TLR-8*) polymorphic site, mutant allele T was prevalent among RA and OA individuals as compared to controls. Two intronic novel mutations were also identified of about 50bp G>C and 39bp T>A before the rs3764879 (*TLR-8*) polymorphic site. The novel SNP's G>C and T>A identified through sequencing were submitted to ClinVar NCBI for obtaining accession numbers SCV000844945 and SCV000844946, respectively. Allele C replaced allele Ton rs1800629 (*TNF*) polymorphic site in both cases whereas on rs361525 (*TNF*) polymorphic site allele C was found to be prevalent among patients as well as controls. No mutation exists on rs2234693 (*ESR-1α*) and rs9340779 (*ESR-1α*), i.e., allele G was found to be significant among patients as well as in controls whereas on rs2228480 (*ESR-1α*) allele G was more prevalent in patients as compared to controls. Similarly, on the rs1451501590 (*ESR-1α*) site, allele G replaced allele C in patients. Two novel mutations were also identified, i.e., SCV000804801 (23G>A) and SCV000804802 (241T>A).

All SNPs followed HWE (*p* = 1.00), and it was observed that in RA individuals except for rs3764879 (*TLR-8*) allelic frequency of rs3764880 (*TLR-8*), rs5744080 (*TLR-8*), SCV000844945 (*TLR-8*), and SCV000844946 (*TLR-8*), polymorphic sites were not significantly varied in comparison to controls. However, in OA cases, except for SCV000844945 (*TLR-8*), rest of the SNPs were not significantly associated with the onset of disease at the allelic level. As a result of the genotypic analysis, it was observed that except for rs3764879 (*TLR-8*), all other SNPs were significantly associated with the onset of RA and except for rs5744080 (TLR-8) all other SNPs were significant risk factors for OA onset. It was observed that rs1800629 (*TNF*) was not significantly associated with the onset of disease at an allelic level; however, it is significantly associated at the genotypic level. In RA, subjects rs2228480 (*ESR-1α*), SCV000804801 (*ESR-1α*), and SCV000804802 (*ESR-1α*) were significantly associated with the onset of disease at an allelic and genotypic level whereas rs1451501590 (*ESR-1α*) was not found to be a significant risk factor in disease onset. In OA, subjects rs2228480 (*ESR-1α*), rs1451501590 (*ESR-1α*), SCV000804801 (*ESR-1α*), and SCV000804802 (*ESR-1α*) were significantly associated with the onset of disease at both allelic and genotypic levels (Table 2).

**Figure 1:**
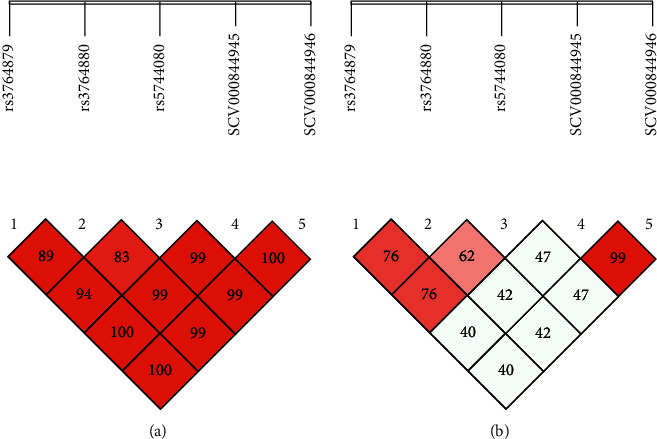
(a, b) Linkage disequilibrium chart of TLR-8 gene polymorphic sites in RA patients and controls.

**Figure 2:**
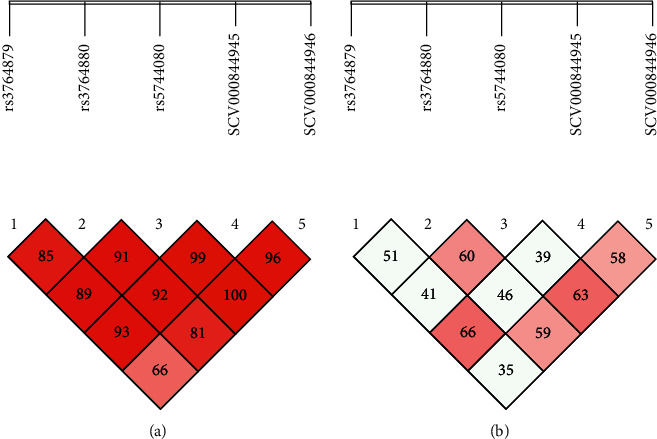
(a, b) Linkage disequilibrium chart of TLR-8 gene polymorphic sites in OA patients and controls.

**Figure 3:**
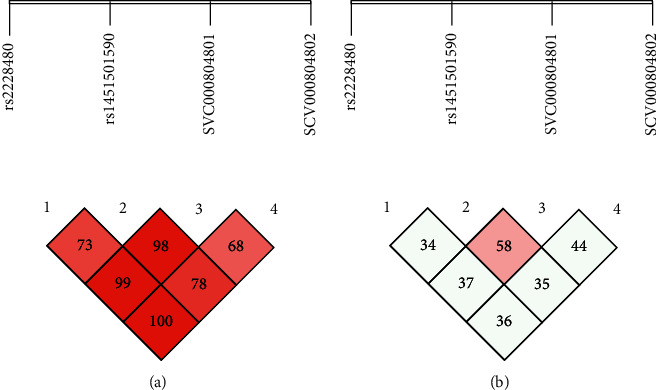
(a, b) Linkage disequilibrium chart of *ESR-1α* gene polymorphic sites in RA patients and controls.

**Figure 4:**
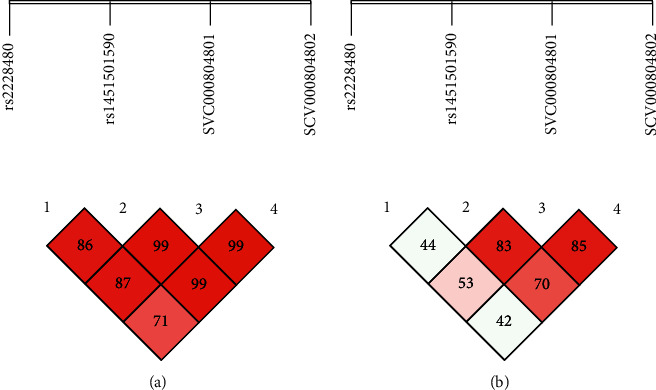
(a, b) Linkage disequilibrium chart of *ESR-1α* gene polymorphic sites in OA patients and controls.

The linkage disequilibrium (L.D) for RA and OA is presented in Figures 1(a), 1(b), 2(a), and 2(b). As a result of L.D, it was observed that in both RA and OA individuals, all SNPs together were significant risk factors with the onset of disease as D' = 1.000; *r*2 = 0405 and D' = 0.660; *r*2 = 0.350. It was observed that SCV000844945 and SCV000844946 were also significant risk factors for the next generation in the onset of disease. As no mutation was observed at rs2234693 and rs9340779 polymorphic sites, therefore, they were excluded from multiple site analyses. As a result of L.D for RA subjects, it was observed that rs2228480, rs1451501590, SCV000804801, and SCV000804802 together were 100% disease onset risk factors in the next generation as D' = 1.000 and *r*2 = 0.360 whereas it also increased the risk of OA onset up to 71% in next-generation D' = 0.717; *r*2 = 0.423. The L.D for ESR-1 is presented in Figures 3(a) and 3(b) for RA and Figures 4(a) and 4(b) for OA, respectively.

On the TLR-8 gene, it was shown that all haplotypes were significant (*p* < 0.01) for the start of disease because their frequency was higher in patients compared to control participants. This was true for both RA and OA. However, the frequency of CACGT and CATGT was higher in controls; therefore, they act as protectants in the onset of disease. *ESR-1α* haplotype analysis indicated that the frequency of AAGT was higher in controls as compared to patients; therefore, it acts as a protectant in RA and OA onset. The significant haplotype in disease onset is presented in Table 3. Change in amino acid sequences with respective polymorphic sites is presented in Table 4.

## 4. Discussion

RA and OA are the two major types of arthritis characterized as multifactorial disorders such as age, BMI, autoimmunity, hormonal, environmental, and genetics which play role in their development and progression. iEssential information has been developed concerning the genome-wide causative mutations and their inheritance through multiple techniques including genome-wide association studies (GWASs), candidate gene association studies Twin, linkage analysis, segregation analysis, and twin studies [28]. Genetic association studies in different populations evidenced that disease development and progression risk are complex processes that might interact with innate as well as acquired host responses to inflammatory, biomechanical, hormonal, environmental, and immunological stimuli [29]. Therefore, the current study targeted for the first time to determine the association of *TLR-8*, *TNF*, and *ESR-1α* gene polymorphism with the onset of not only RA but also OA in the Pakistani population.

Currently, the study demonstrated that rs3764879 of *TLR-8* was associated with RA onset at an allelic level whereas rs3764880 and rs5744080 were associated at the genotypic level. In OA development, rs3764879 and rs3764880 were significant risk factors genotypically. Two novel functional mutations never reported before, i.e., SCV000844945 and SCV0008449456 were also identified on *TLR-8* and found to be more associated with OA development as compared to RA onset. Overall polymorphism on the *TLR-8* gene was found to be significant in disease onset. Like current findings, a replicate study reported the linkage of RA onset and rs3764880 polymorphism in the Caucasian Spanish population. In addition, signaling of *TLR-8* induced pro-inflammatory cytokines [30]. Polymorphism on rs3764880 leads to a decrease in inflammatory cytokines levels. This polymorphism in the start codon causes *TLR-8*'s first three amino acid deletions giving rise to the TLR8v2 isoform [31]. A study conducted on OA Chinese individuals reported rs5744080 polymorphisms as a significant risk factor in the onset of disease in male subjects [32]. It is well documented that variants on the *TLR-8* genes were associated with autoimmune and infectious diseases like systemic lupus erythematosus, type 2 diabetes, and tuberculosis [33–35]. However, more studies should be conducted to determine its association with RA and OA development.

The current study revealed a strong association of Rs1800629 polymorphic site variation with both RA and OA development. In the pipeline of current findings, the same functional polymorphism rs1800629 with RA as well as OA onset was also reported in the north Indian population, Chinese population, and Egyptian population [36–38]. In contrast, no association between rs1800629 and RA was reported in the Brazilian population [39]. On the other hand, the current study reported no association between rs361525 and disease development. However, contrary to current findings, another study conducted on the Pakistani population reported a positive association between rs361525 and OA development in the Pakistani population [40]. Like the findings of the current study, no positive association has been reported in the Chinese, Mexican, and Iraqi populations [40–42].

It was declared from the present study that no association exists between rs2234693 and rs9340779 with the onset of both RA as well as OA in the studied population. The current study reported that polymorphism on rs2228480 polymorphic site acts as a significant risk factor in the onset of both RA and OA. It was also revealed that rs1451501590 polymorphism was associated with OA development only. Although the vital role of estrogen in the pathogenesis of RA was indicated by many studies, no significant effects were reported by estrogen receptor agonists on RA symptoms [43]. Van Vollenhoven [44] reported a lack of clinical advantages of selective *ESR-1α* agonist treatment in RA patients. Like current findings, no association was reported between RA and rs2234693 and rs9340779 polymorphic sites in the polish and Japanese populations [43]. Studies conducted in Caucasian, European, and American populations reported a non-significant association between rs2234693 and 9340779 and RA [45, 46]. In contrast to current findings, strong association of rs2234693 and rs9340779 with OA was reported in Japan, Korean, and Mexican populations [47, 48]. A meta-analysis conducted by Hu et al [49] reported that polymorphism on rs2234693 might reduce the risk of OA risk and the rs9340799 may not be associated with OA risk in the Chinese population. A weak association relationship in rs9340799and OA was reported in Europeans but not Asians whereas rs2234693 was not significantly associated with OA in both populations [50]. Another study conducted by Ma et al. [51] reported that the risk of OA incidence decreased with rs9340799 and rs2228480 whereas rs224693 was a strong factor in disease onset. Jin et al. [52];reported in their case-control study that the minor allele of rs2228480 was an increased risk of knee OA in his Meta-analysis, indicated the same results. The current study also reported the identification of two significant risk SNPs, i.e., SCV000804801 and SCV000804802. SCV000804802 was a functional novel SNP and caused the replacement of phenylalanine with isoleucine.

*ESR-1α* polymorphisms altered the estrogen receptor expression in the *ESR-1*, and *ESR-2* genes could affect the expression that leads to immunological consequences. The effect of estrogen was mediated by estrogen receptors located in the multiple immune cells (T cells, B cells, monocytes, and macrophages) as well as in the thymus [53]. This effect can be achieved by directly manipulating the profile of T-helper cytokine from interleukin (IL)-2, *TNF*-*α*, and interferon (IFN)-*γ* (pro-inflammatory) to IL-4, IL-6, and IL-10, transforming growth factor (TGF)-*β* (anti-inflammatory) direction [54].

The current study concluded that *TLR-8* and *ESR-1α* gene polymorphism are the significant risk factors in the onset of both RA as well as OA individuals in the Pakistani population. However, larger scale studies in other populations should be conducted to determine novel mutation susceptibility.

## Figures and Tables

**Table 1:** PCR-RFLP materials and conditions with respective polymorphic sites.

Gene	SNP ID	F.P	R.P	A.T (^o^C)	Product size (bp)	RFLP Enzyme	I.T (^o^C)	T.D.T (^o^C)	Fragments size (bp)
TLR-8	rs3764879	GTGTGTGTCTGATTTGGGTTG	TTTCTAGGCTCACACCATTTG	58	390	HpyCh4IV	65	—	CC: 390 CG: 390, 228, 162 GG:228, 162
	rs3764880					NlaIII	37	65	AA: 39 AG: 390, 291, 990 GG: 291, 99
	rs5744080	CCCCAAATACCCTCTGGTTT	ATTGAAGCACCACCATCACA	58	416	HpyCh4IV	65	—	CC: 416 CT: 416, 234, 182 TT: 234, 182

*TNF*	Rs1800629	AGGCAATAGGTTTTGAGGGGCAT	ATCTGGAGGAAGCGGTAGTG	59.5	226	NcoI	37	65	AA: 226 AG: 226, 202 GG: 202
	rs361525					MspI	37	65	GG: 226

*ESR-1α*	rs2234693	CTGCCATCTTTTCCTATTCTCC	TCTTTCTCTGCCACCCTGGCGTCGATTATTATCTGA	59	636	PuvII	37	80	CC: 636
	rsS9340779					XbaI	37	65	CC: 636
	rs2228480	GTGGAGGAGACGGACCAAA	TGGCCACTCATCTAGAAAGCC	60.5	246	BtgI	37	80	AA: 246 AG: 246, 145, 101 GG: 145, 101
	rs1451501590					—	—		—

F.P: forward primer; R.P: reverse primer; A.T: annealing temperature; RFLP: restriction fragment length polymorphism; I.T: incubation temperature; T.D.T: thermal deactivation temperature.

**Table 2:** Single site analysis of all targeted polymorphic sites in RA as well as OA subjects.

Gene	Allele/genotype	RA frequency (P/C)	*X* ^2^ value	*p* value	OA frequency (P/C)	*X* ^2^ value	*p* value
TLR-8	Rs3764879						
	C	0.057/1.000	1290.075	0.002^*∗*^	0.361/1.000	729.016	>0.01
	G	0.943/0.000			0.639/0.000		
	GG	0.887/0.000	711.999	>0.01	0.278/0.000	727.999	0.002^*∗*^
	CG	0.113/0.000			0.722/0.000		
	CC	0.000/1.000			0.000/1.000		
	Rs3764880						
	A	0.088/1.000	1219.788	>0.01	0.196/1.000	1017.99	>0.01
	G	0.912/0.000			0.804/0.000		
	GG	0.823/0.000	712.000	0.001^*∗*^	0.608/0.000	728.000	0.001^*∗*^
	AG	0.177/0.000			0.392/0.000		
	AA	0.000/1.000			0.000/1.000		
	Rs5744080						
	C	0.170/0.981	988.959	>0.01	0.038/0.981	1297.66	>0.001
	G	0.830/0.019			0.962/0.019		
	TT	0.660/0.000	655.964	0.002^*∗*^	0.924/0.000	688.920	>0.01
	CT	0.340/0.039			0.076/0.039		
	CC	0.000/0.961			0.000/0.961		
	SCV000844945						
	C	0.500/0.000	521.964	>0.01	0.524/0.000	558.531	0.001^*∗*^
	T	0.500/1.000			0.476/1.000		
	CC	0.000/0.000	712.000	0.001^*∗*^	0.047/0.000	728.000	0.001^*∗*^
	CG	1.000/0.000			0.953/0.000		
	GG	0.000/1.000			0.000/1.000		
	SCV000844946						
	A	0.500/0.000	521.964	0.000	0.741/0.000	899.208	>0.01
	T	0.500/1.000			0.259/1.000		
	AA	0.000/0.000	712.000	0.001^*∗*^	0.481/0.000	727.999	0.002^*∗*^
	AT	1.000/0.000			0.519/0.000		
	TT	0.000/1.000			0.000/1.000		

*TNF*	Rs1800629						
	G	0.722/0.000	753.867	>0.01	0.910/0.000	1023.640	>0.01
	A	0.228/1.000			0.090/1.000		
	GG	0.543/0.000	600.000	0.001^*∗*^	0.820/0.000	616.000	0.002^*∗*^
	AG	0.457/0.000			0.180/0.000		
	AA	0.000/1.000			0.000/1.000		
	Rs361525						
	G	1.000/1.000	—	—	1.000/1.000	—	—
	GG	1.000/1.000	—	—	1.000/1.000	—	—

*ESR-1α*	Rs2234693						
	C	1.000/1.000	—	—	1.000/1.000	—	—
	CC	1.000/1.000	—	—	1.000/1.000	—	—
	Rs93407779						
	C	1.000/1.000	—	—	1.000/1.000	—	—
	CC	1.000/1.000	—	—	1.000/1.000	—	—
	Rs2228480						
	A	0.127/1.000	930.177	0.002^*∗*^	0.286/1.000	675.414	0.002^*∗*^
	G	0.873/0.000			0.714/0.000		
	AA	0.000/1.000	600.000	0.002^*∗*^	0.073/1.000	530.493	0.002^*∗*^
	AG	0.253/0.000			0.427/0.000		
	GG	0.747/0.000			0.500/0.000		
	Rs1451500						
	A	0.342/1.000	588.819	>0.01	0.509/1.000	393.256	0.001^*∗*^
	G	0.658/0.000			0.491/0.000		
	AA	0.057/1.000	535.645	>0.01	0.041/1.000	566.126	0.001^*∗*^
	AG	0.570/0.000			0.937/0.000		
	GG	0.373/0.000			0.022/0.000		
	SCV000804801						
	A	0.455/0.000	353.398	0.001^*∗*^	0.562/0.000	473.449	0.001^*∗*^
	G	0.545/1.000			0.438/1.000		
	AA	—	500.917	0.001^*∗*^	0.123/0.000	616.000	0.002^*∗*^
	AG	0.910/0.000			0.877/0.000		
	GG	0.090/1.000			0.000/1.000		
	SCV000804802						
	A	0.437/0.000	335.181	0.002^*∗*^	0.628/0.000	564.014	0.001^*∗*^
	T	0.563/1.000			0.372/1.000		
	AA	0.130/0.000	354.907	0.001^*∗*^	0.256/0.000	616.000	0.002^*∗*^
	AT	0.613/0.000			0.744/0.000		
	TT	0.257/1.000			0.000/1.000		

**Table 3:** Haplotype analysis of TLR-8 and *ESR-1α* gene.

Haplotype	RA frequency (P/C)	*p* value	Haplotype	OA frequency (P/C)	*p* value
Haplotype: rs3764879, rs3764880, rs5744080, SCV000844945, SCV000844946					
TLR-8					
CACGT	0.000/0.981	0.006^*∗*^	CACGT	0.000/0.981	0.001^*∗*^
CATGT	0.000/0.019	0.0005^*∗*^	CATGT	0.000/0.019	0.0004^*∗*^
CGCCA	0.014/0.000	0.005^*∗*^	CATCA	0.075/0.000	0.001^*∗*^
CGCCT	0.014/0.000	0.005^*∗*^	CATCT	0.040/0.000	0.009^*∗*^
CGCGA	0.014/0.000	0.005^*∗*^	CGTCA	0.176/0.000	0.001^*∗*^
CGCGT	0.014/0.000	0.0005^*∗*^	CGTCT	0.070/0.000	0.001^*∗*^
GATCA	0.013/0.000	0.001^*∗*^	GATCA	0.013/0.000	0.009^*∗*^
GATCT	0.018/0.000	0.001^*∗*^	GATCT	0.068/0.000	0.001^*∗*^
GATGA	0.018/0.000	0.001^*∗*^	GGCCT	0.038/0.000	0.008^*∗*^
GTTGT	0.018/0.000	0.001^*∗*^	GGTCT	0.043/0.000	0.009^*∗*^
GGCCA	0.018/0.000	0.0006^*∗*^	GGTGC	0.476/0.000	0.002^*∗*^
GGCCT	0.024/0.000	0.0006^*∗*^			
GGCGA	0.024/0.000	0.006^*∗*^			
GGCGT	0.024/0.000	0.0006^*∗*^			

*Haplotype: rs2228480, rs1451501590, SCV000804801, SCV000804802*					
*ESR-1α*					
AAGT	0.025/1.000	0.002	AAGT	0.000/1.000	0.002
AAAT	0.003/0.000	—	AAAA	0.218/0.000	0.002
AGGT	0.098/0.000	0.001	AGAA	0.005/0.000	—
GAGA	0.311/0.000	0.001	AGGA	0.064/0.000	0.001
GAGT	0.002/0.000	—	AGGT	0.000/0.000	—
GGAA	0.067/0.000	0.001	GAAA	0.292/0.000	0.001
GGAT	0.385/0.000	0.001	GGAA	0.047/0.000	0.008
GGGA	0.059/0.000	0.009	GGGA	0.003/0.000	—
GGGT	0.050/0.000	0.008	GGGT	0.372/0.000	0.002

**Table 4:** Change in amino sequence by MEGA 6.0 software.

Gene	SNP ID	Wild amino acid	Mutant amino acid
TLR-8	Rs3764880	Valine	Methionine
	Rs5744080	Histamine	Histamine
	SCV000844945	Arginine	Threonine
	SCV0008844946	Phenylalanine	Isoleucine
*TNF*	Rs1800629	Upstream transcript variant	
	Rs361525	Upstream transcript variant	
ER1-*α*	Rs2228480	Thrionine	Thrionine
	SCV000804802	Phenylalanine	Isoleucine
	Rs1451501590	3 prime UTR variant	

## Data Availability

The data used to support the findings of this study are available from the corresponding author upon request.
